# Compression Molding of Thermoplastic Polyurethane Foam Sheets with Beads Expanded by Supercritical CO_2_ Foaming

**DOI:** 10.3390/polym13040656

**Published:** 2021-02-22

**Authors:** Tao Zhang, Seung-Jun Lee, Yong Hwan Yoo, Kyu-Hwan Park, Ho-Jong Kang

**Affiliations:** 1 Department of Polymer Science and Engineering, Dankook University, 152 Jukjeon-ro, Suji-gu, Yongin-si, Gyeonggi-do 16889, Korea; taozhang1214@gmail.com; 2 HDC Hyundai EP R&D Center, 603 Graduate Schools Bldg., Dankook University, 152 Jukjeon-ro, Suji-gu, Yongin-si, Gyeonggi-do 16889, Korea; jjun1984@hdc-hyundaiep.com (S.-J.L.); yooyh@hdc-hyundaiep.com (Y.H.Y.); kyu@hdc-hyundaiep.com (K.-H.P.)

**Keywords:** thermoplastic polyurethane, expanded bead, supercritical CO_2_ foaming, expansion ratio, resilience, hardness

## Abstract

Expanded thermoplastic polyurethane (ETPU) beads were prepared by a supercritical CO_2_ foaming process and compression molded to manufacture foam sheets. The effect of the cell structure of the foamed beads on the properties of the foam sheets was studied. Higher foaming pressure resulted in a greater number of cells and thus, smaller cell size, while increasing the foaming temperature at a fixed pressure lowered the viscosity to result in fewer cells and a larger cell size, increasing the expansion ratio of the ETPU. Although the processing window in which the cell structure of the ETPU beads can be maintained was very limited compared to that of steam chest molding, compression molding of ETPU beads to produce foam sheets was possible by controlling the compression pressure and temperature to obtain sintering of the bead surfaces. Properties of the foam sheets are influenced by the expansion ratio of the beads and the increase in the expansion ratio increased the foam resilience, decreased the hardness, and increased the tensile strength and elongation at break.

## 1. Introduction

Polymer foams [1,2] are widely used for light weight polymer molded products. Typical processes for making light weight polymer molded products are the Mucell process [3,4] and the bead foam process [5,6]. In the Mucell process, chemical foaming agents [7,8] or physical foaming agents [9,10,11] are added to the polymer melt and the melt is transferred through the die or into the mold under pressure and cooled in the extrusion or injection molding process. In the bead foam process, expanded beads [12,13] which have already been foamed or expandable beads [14] containing foaming agents which can be foamed are used to prepare foamed products. Incorporation of the foaming agent into the polymer pellet can be carried out by addition in the polymerization process [15,16], by using a high temperature and pressure autoclave to introduce the foaming agent in the supercritical fluid state to polymer pellets [17,18], or by adding the foaming agent to the polymer melt in the extruder and preparing expandable or expanded beads by controlling the cooling condition [19].

Expandable beads are used most widely in the case of polystyrene [20], and expanded beads are used in the case of polypropylene (expanded polypropylene, EPP) [21], polystyrene (expanded polystyrene, EPS) [22], and polyethylene (expanded polyethylene, EPE) [23]. Expanded bead foams are manufactured through a sintering process using foamed polymer beads, which have excellent insulation, heat resistance, impact resistance, and energy absorption. In particular, EPP is widely used for light weight automobile parts due to its mechanical properties, low thermal conduction, and shock absorption properties [24,25]. Recently, interest in expanded thermoplastic polyurethanes (ETPU), which can be used to prepare soft and flexible material and whose properties can easily be controlled in the polymerization process, is increasing [26,27]. These thermoplastic polyurethane foams are excellent flexible materials with high hardness, rebound resilience, excellent mechanical properties, and dynamic shock absorption. In manufacturing polymer foam molded products from expanded beads, especially in the case of EPP, steam chest molding is used [28,29], where high temperature steam is fed into the injection mold to physically sinter and bond the bead surfaces. The critical factor in this process is maintaining the cell structure of EPP while bonding the bead surfaces, thus the temperature of the steam, pressure, and residence time are important variables. Along with the research and development of expanded beads, research on steam chest molding of expanded beads has been reported [30]; the incorporation of hot air along with steam for uniform penetration of the steam to reduce the molding defects from steam variation has also been reported [31]. Steam chest molding is indisputably the best process for molding of expanded beads, but due to high equipment costs, its general applicability is limited and thus, research on diverse methods to fabricate molded foam products appears to be required.

Compression molding was utilized in this study to diversify the methods for fabricating foam products, as it is the most typical and inexpensive fabrication method in polymer processing. Foam sheets were prepared from expanded thermoplastic polyurethane (ETPU) foamed under diverse supercritical CO_2_ foaming conditions, and the effect of bead foam structure on the characteristics of the foam sheets was studied.

## 2. Materials and Methods

**Figure 1:**
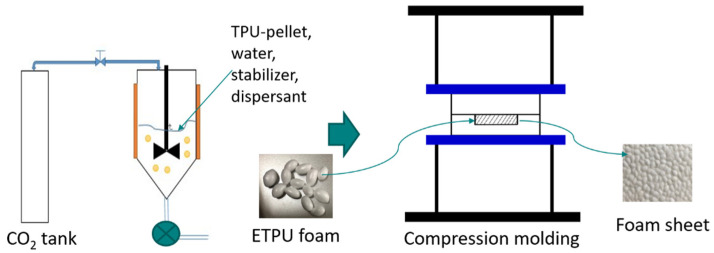
Schematic of the CO_2_ assisted foaming process for preparing expanded thermoplastic polyurethane (ETPU) beads and the compression molding process.

The thermoplastic polyurethane used in this study was Dongsung Corp. (Busan, Korea) aromatic polyether thermoplastic polyurethane (TPU: 6175AP), having a melting point of 150 °C, specific gravity of 1.055 g/cm^3^, and Shore A hardness of 78. A lab-designed autoclave (CRS, Anyang, Korea) was used for the foaming of TPU to prepare the ETPU beads. The autoclave was charged with 250 g distilled water, 100 g TPU, 6.70 g tricalcium phosphate (TCP, Sigma-Aldrich, Merck KGaA, Darmstadt, Germany) stabilizer, and 0.13 g sodium dodecylbenzenesulfonate (SDBS, Sigma-Aldrich, Merck KGaA, Darmstadt, Germany) dispersing agent, then CO_2_ was pumped in with a high-pressure pump (CRS, Anyang, Korea). In order to obtain supercritical CO_2_, the temperature was set at 90, 100, 105, or 110 °C and the pressure was set at 75, 80, or 90 bar; the TPU was kept in the autoclave for 30 min, then the pressure was quickly released to atmospheric pressure by opening a ball valve to prepare expanded TPU (ETPU) beads. To prepare TPU foam sheets, a mold cavity measuring 10 cm × 10 cm × 2.0 mm with a temperature control system was mounted on a compression molding machine (QMESYS, QM900A, Uiwang, Korea). Foam sheets were prepared by keeping 15 g ETPU charged mold at 140–150 °C and 3.5–10.5 MPa for 2–15 min to sinter the bead surfaces then quenching in water at 4 °C. A schematic of the foaming process to prepare the ETPU beads and the foam sheet compression molding process is shown in Figure 1.

The water displacement method used to measure the density of all samples was according to ASTM-D792. The foam structure of the ETPU beads prepared under different temperature and pressure conditions was characterized by measuring the cell diameter (D) and the cell density (N) using micrographs obtained with a scanning electron microscope (Coxem EM-30, Daejeon, Korea). The expansion ratio was determined by measuring the density of the pellet before and after foaming (*ρ*_TPU_, *ρ*_ETPU_) using an electronic densitometer (SD-200L, Vaughan, ON, Canada) then calculating the expansion ratio (Φ) using the following equation.

Φ = *ρ*_TPU/_*ρ*_ETPU_ (1)

Five ETPU foam sheet samples with sizes of 20 mm × 90 mm × 3 mm were prepared for tensile testing at the speed of 10 mm/min. The mechanical properties of the prepared ETPU foam sheets were evaluated by measuring the tensile strength, modulus, and elongation at break as a function of extension ratio using a tensile tester (Lloyd LR30K, Cleveland, OH, USA) and measuring the Shore A hardness using a Shore hardness tester (BS-392-A, Guangzhou Amittari Instruments Co., Ltd., Guangzhou, China). The rebound properties of the foam sheets were evaluated by dropping a 5 mm ball weighing 0.486 g from 41 cm height (*H*_o_) and measuring the height it rebounded (*H*) with a lab-made rebound tester; the ball rebound ratio was calculated according to the following equation.

*R* = *H*/*H*_o_ × 100(%) (2)

## 3. Results and Discussion

**Figure 2:**
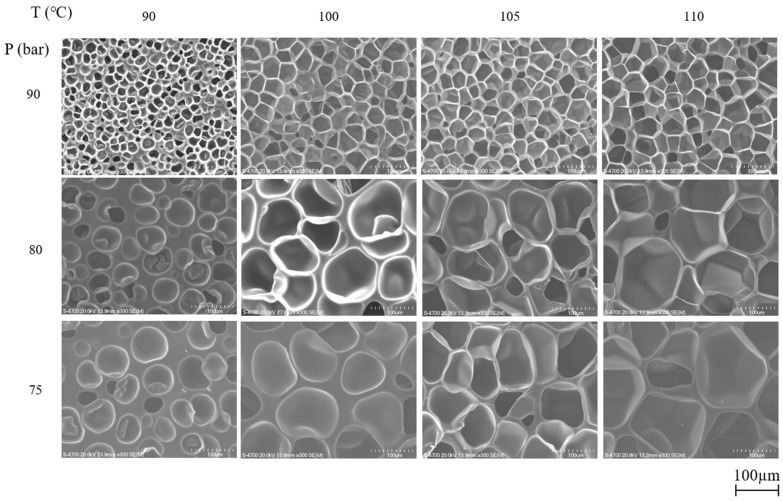
SEM micrographs of ETPU prepared at different foaming temperatures and pressures in the supercritical CO_2_ foaming process.

**Figure 3:**
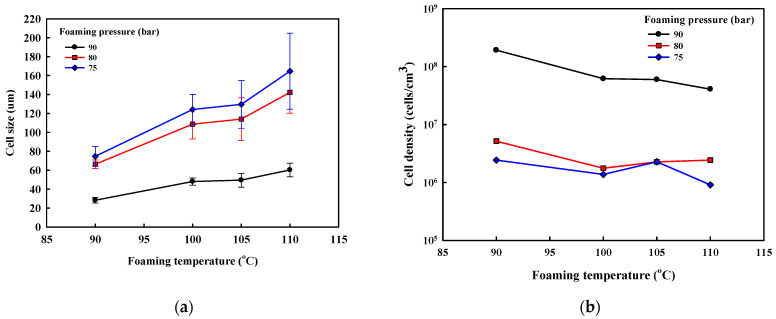

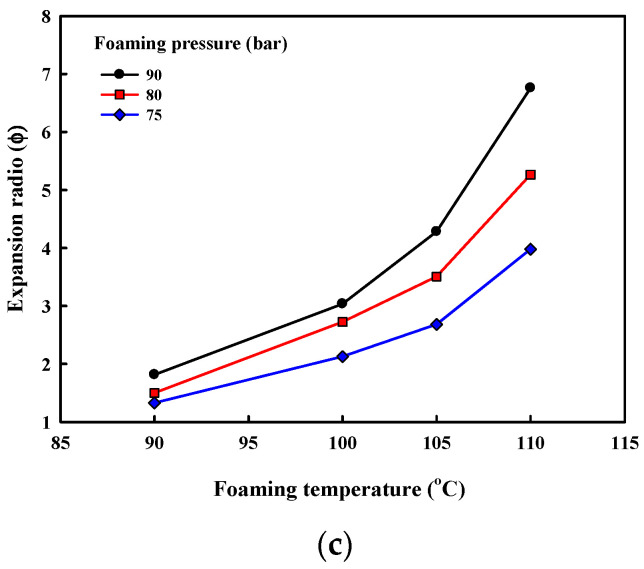
Physical properties of ETPU beads prepared at different foaming temperatures and pressures in the supercritical CO_2_ foaming process: (**a**) foam size; (**b**) foam density; (**c**) expansion ratio.

The SEM micrographs of ETPU prepared under different foaming temperatures and pressures are shown in Figure 2. The cell diameter and density measured from Figure 2, and the expansion ratio determined from the density measurements of the pellet before and after foaming shown in Figure 3, reflect the effect of the foaming temperature and pressure on these values. It can be seen in Figure 2 that under the temperature and pressure conditions used in this study, the ETPU foam has a closed cell structure. As can be seen in Figure 2, when the pressure is low (75 bar), the cell is not fully developed and the walls between cells are thick, suggesting that the condition is not adequate for preparing ETPU. The cell size decreases with the rise in pressure and at 90 bar, the cell diameter is 20–60 μm and the cell density is 10^8^ cells/cm^3^, allowing it to be classified as a fine cell foam [32], regardless of the temperature. In contrast, below 90 bar, the cell diameter is greater than 100 μm and the cell density is 10^6^ cells/cm^3^, representative of conventional cell foam. This is a result of more nuclei being formed in the TPU at higher pressures, where the same total amount of CO_2_ is subsequently diffused and the expansion occurring therefrom forms relatively smaller cells. At a fixed pressure, a temperature increase decreases the viscosity of TPU and results in larger cells and lower cell density. The expansion ratio increases with the increase in the pressure and temperature of the foaming process, suggesting that it is more dependent on the cell size compared with cell density. As can be seen in Figure 3c, the expansion ratio of most ETPU obtained in this study is generally below 4, characteristic of high-density foams. However, when the foaming is carried out at 80–90 bar and 110 °C, medium-density foams characterized by expansion ratios of 4–10 are obtained, and when the foaming is carried out at 90 bar and 110 °C, the highest expansion ratio of 7 is obtained. The foam structure, which is dependent on the foaming conditions, will no doubt affect the properties of the foam sheets made from ETPU beads.

**Figure 4:**
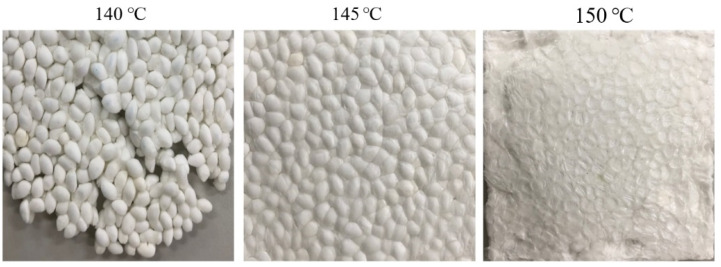
Effect of molding temperature in the compression molding of ETPU beads at 3.5 MPa for 15 min.

**Figure 5:**
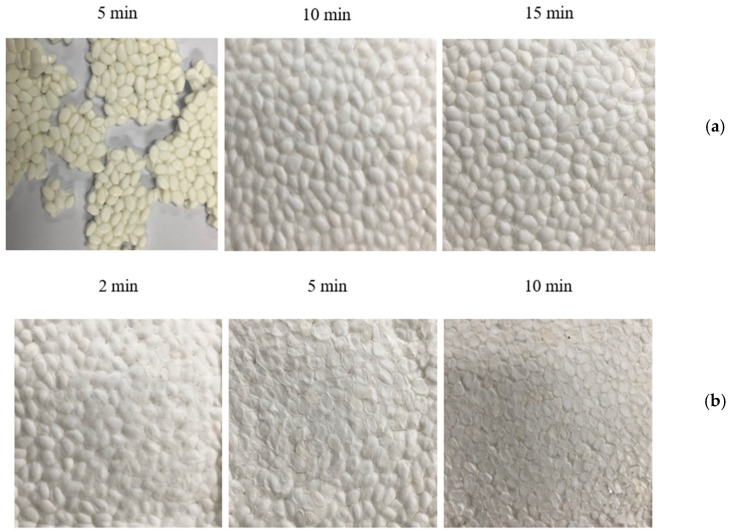
Effect of molding time in the compression molding of ETPU beads at 3.5 MPa, molding temperature: (**a**) 145 °C; (**b**) 150 °C.

**Figure 6:**
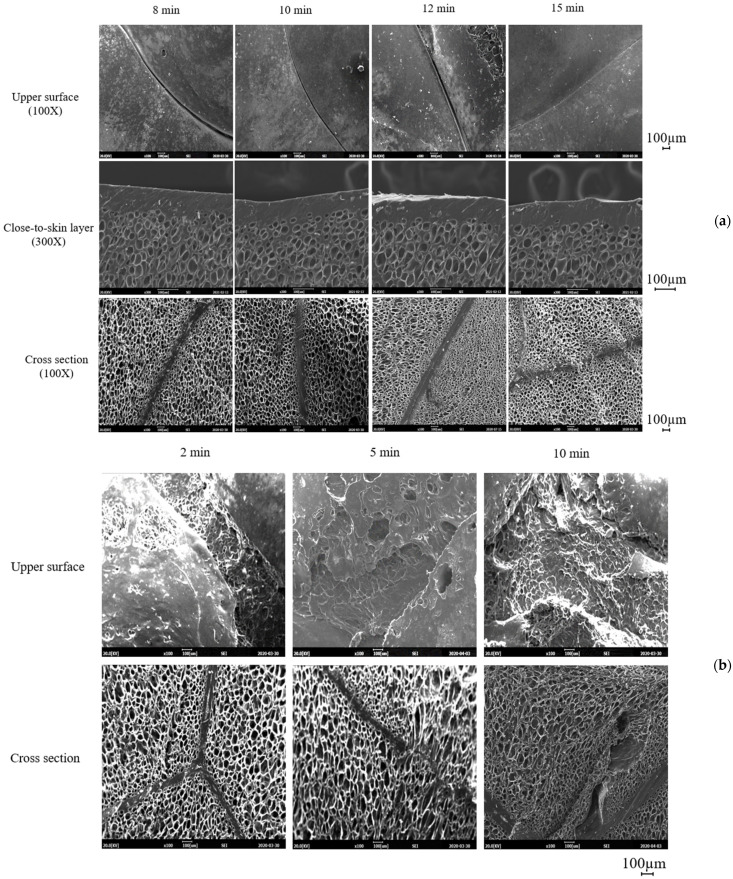
SEM micrographs of the surface and cross section of ETPU sheets molded at (**a**) 145 °C and (**b**) 150 °C.

The effect of the molding temperature on the structure of foam sheets prepared by a 15 min compression molding of ETPU beads at 105 °C and 90 bar can be seen in Figure 4. As can be seen, when compression molded at 140 °C, the fabrication of foam sheets is not possible as sintering does not occur sufficiently, while at 150 °C, melting of the surface of the beads occurs, suggesting that preparation of foam sheets by compression molding should be carried out in a narrow range of temperature slightly below 150 °C, which is the melting point of TPU. The effect of molding time on the formation of the foam sheets at 145 and 150 °C is shown in Figure 5. At 145 °C, sintering of the beads does not occur in 5 min as in the case of molding at 140 °C, but occurs sufficiently in 8–15 min without deformation of the cells. At 150 °C, foam sheets maintaining the bead structure are formed when the molding time is relatively short at 2–3 min; however, at longer molding times, deformation of the sheet surface can be seen contrary to those molded at 145 °C. Surface and cross section SEM micrographs of the samples, prepared under the same conditions as in Figure 5, are shown in Figure 6. The surface of the foam sheet molded at 145 °C in Figure 6a is smooth and does not show irregular surface melting of the TPU, but that molded at 150 °C in Figure 6b shows irregular surface melting and consequently, destructive deformation of the surface. It seems that similar cell morphology was obtained between the core and the close-to-skin layer. Under both conditions, the interface between the beads becomes thicker with molding time, suggesting effective sintering of the bead surfaces. Although there is no deformation of the cell structure when molded at 145 °C, cell deformation from the original ETPU occurs at 150 °C with an increase in molding time due to melting, especially at the interface between beads where interfacial sintering occurs.

**Figure 7:**
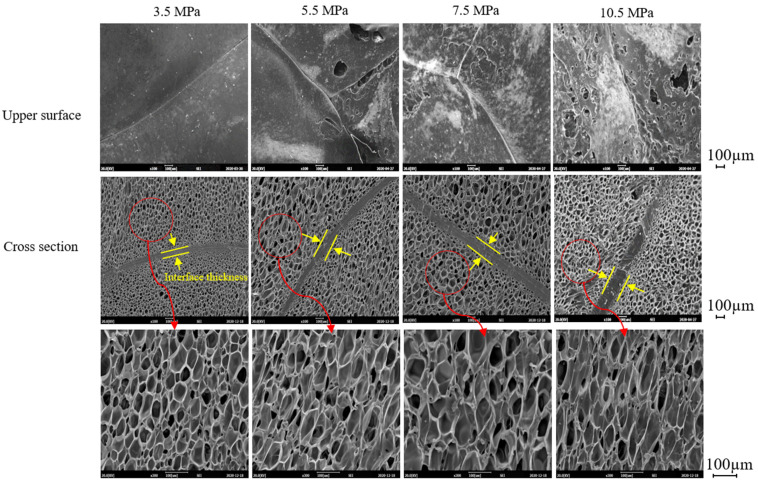
Effect of compression molding pressure on the sintering of ETPU beads at 145 °C.

The effect of compression molding pressure on the sintering of beads is shown in Figure 7. The interface between beads becomes thicker with the increase in pressure which may increase the physical properties of the foam sheets; however, destructive deformation of the foam surface and cell deformation near the interface can be seen as in the case of increasing molding times (Figure 6). Thus, compression molding at 3.5 MPa appears to result in the best foam sheets. Based on these results, compression molding of ETPU foam sheets is possible, but when compared with steam chest injection molding, the temperature and pressure range at which cell deformation can be minimized is very limited and thus, precise control of the molding temperature and pressure is required.

**Figure 8:**
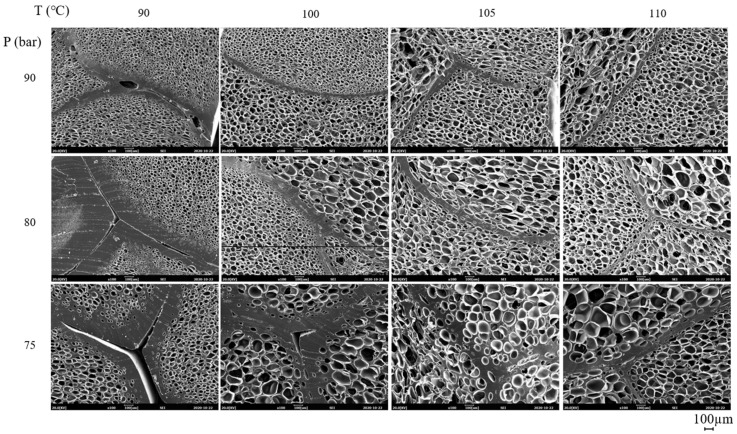
SEM micrographs of cross section of ETPU foam sheets made by compression molding at 3.5 MPa and 145 °C for 15 min.

The effect of cell structure on the properties of the foam sheets is studied by compression molding ETPU beads, prepared under different conditions and thus, having different cell structure, at 3.5 MPa and 145 °C for 15 min, which is the molding condition where sintering of the beads occurs and deformation of the cell can be minimized. The SEM micrographs of foam sheets compression molded at 145 °C for 15 min with ETPU having different cell diameter and cell density are shown in Figure 8. In all ETPU, sintering through surface fusion was sufficient; when the ETPU foamed at low pressure and temperature is used, the interface formed by fusion of the beads is thicker and the cell structure is not deformed in the compression molding process.

**Figure 9:**
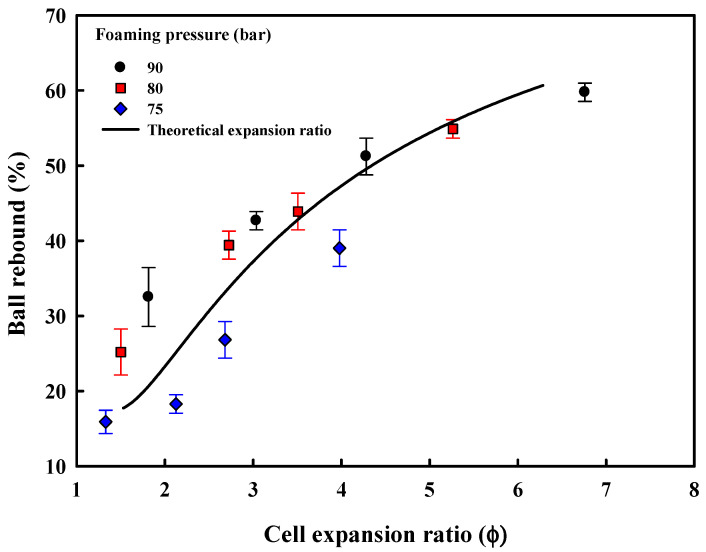
Effect of foam structure on the ball rebound of ETPU foam sheets.

The effect of the cell diameter, cell density, and expansion ratio on the rebound properties of the compression molded foam sheets is shown in Figure 9. The ball rebound property is generally used to evaluate the resilience of foams. Unlike hardness, the ball rebound property reflects the instantaneous feel of the foam and when the foam has poor resilience or low energy absorption, it exhibits lower rebound. The rebound property is generally controlled by appropriate selection of the isocyanate and polyol used in the polymerization of the polyurethane. However, as can be seen in Figure 9, even with a single polyurethane different ball, rebound properties can be obtained by compression molding ETPU of different cell structure, obtained by foaming TPU under different conditions. The ball rebound property is dependent on the expansion ratio and is higher in the case of foams having higher expansion ratios (Figure 9), suggesting that medium-density foams have higher foam resilience and energy absorption compared with high-density foams. The foam sheet prepared with ETPU that foamed at 75 bar exhibits a relatively low ball rebound (Figure 9), which appears to be due to the insufficient cell expansion in TPU at the low foaming pressure (Figure 2). The theoretical expansion ratio that can be calculated from the cell volume (*V_g_*), which, in turn, can be calculated from the number of cells (*N*) and the cell diameter (*D*) in Figure 3 and the theoretical expansion ratio (Φ_Theoretical value_) from the pellet volume (*V_p_* = 1), is shown in Figure 9, along with the measured data. It shows a similar correlation with the experimental expansion ratio (Φ) calculated using the measured densities before and after foaming, suggesting the theoretical expansion ratio calculated considering the two mutually complementary factors—cell diameter and cell density—correlates with the properties of the foam sheet.

(3) $$Vg=N\frac{\pi }{6}D^{3}$$

(4) $$\Phi_{\mathrm{Theoretical}\;\mathrm{value}}=\left(V_{p}+V_{g}\right)/V_{p}=1+V_{g}\;=1+N\frac{\pi }{6}D^{3}$$

**Figure 10:**
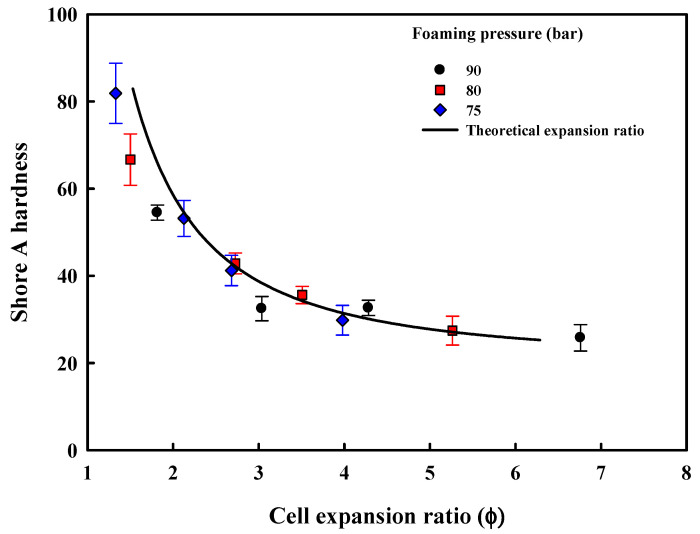
Effect of foam structure on Shore A hardness of ETPU foam sheets.

Figure 10 shows the Shore A hardness of the prepared foam sheets. Contrary to the ball rebound property, foams with a low expansion ratio due to small cell diameter and a small number of cells were relatively hard; on the other hand, foams with higher expansion ratios exhibited low hardness and thus, were soft. The foam sheet prepared with ETPU foamed at low pressure and temperature of 75 bar and 90 °C, where cell expansion was not complete, shows a hardness value similar to the TPU sheet which had not been foamed. This appears to be due to the inadequate foaming condition resulting in a greater portion of the ETPU not being foamed. The hardness showed negligible change with the increase in the expansion ratio above 4, showing that in the case of the medium-density foam sheets (expansion ratio ≥ 4), the expansion ratio determined by the number and size of cells does not affect the hardness of the foam sheets, while it does in the case of high-density foams (expansion ratio < 4).

**Figure 11:**
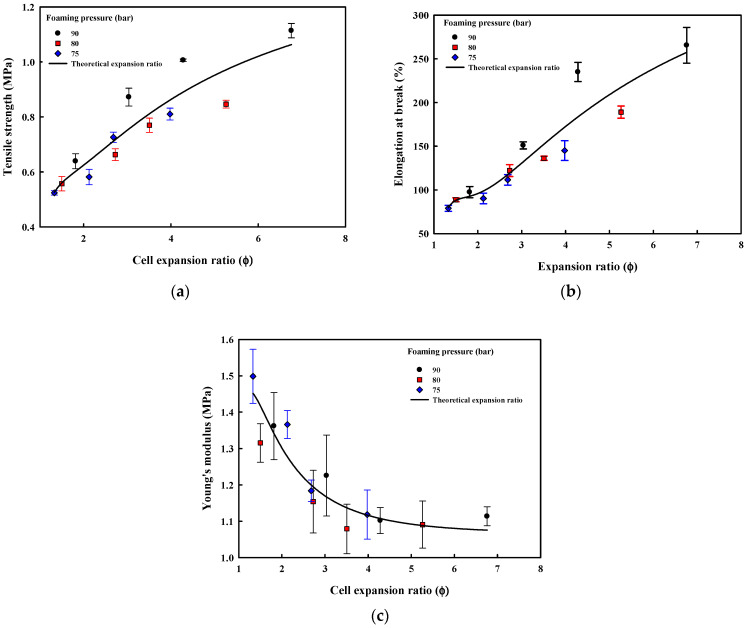
Mechanical properties of ETPU foam sheets: (**a**) tensile strength; (**b**) elongation at break; (**c**) Young’s modulus.

**Figure 12:**
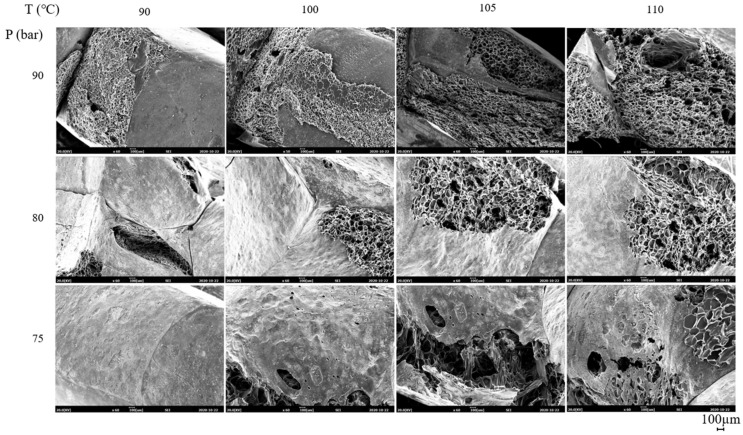
SEM micrographs of the fracture surface of ETPU foam sheets from tensile testing.

The tensile properties of the foam sheets are shown in Figure 11. The tensile strength and elongation at break increase with the increase in the expansion ratio but the modulus decreases. Foam sheets made from ETPU beads foamed at a relatively low pressure and temperature with thick intercell walls and thus, low expansion ratios are ruptured easily at the sintered interface between the beads by the applied tensile force, as the modulus of the parts that have not been foamed is higher. The tensile strength of the foam sheets made from expanded beads is dependent on the failure of the sintered interface between the beads and the failure of the cells inside the beads. When the compression molding conditions are not adequate and sintering is insufficient, failure at the bead interface is expected to result in very poor tensile strengths, but the mechanical properties of the foam sheets processed under appropriate conditions are expected to depend on the failure at the interface or cell depending on the cell structure. In Figure 12, showing the cross-section SEM micrographs of fracture surfaces resulting from tensile testing of the foam sheets processed under optimum compression molding conditions in this study, it can be observed that failure at both the interface and cells occurs with the relative degree depending on the ETPU used. Foam sheets made from low expansion ratio beads fail at the bead–bead interface, while those having higher expansion ratios from higher foaming pressures and temperatures fail at the cell, resulting in higher tensile strengths and elongation at break. That is, the closed cell structure in the beads with high expansion ratios absorbs the energy in tensile testing without failure at the interface until the cell finally fails instead of the interface. Based on these results, it appears that using medium-density foam beads rather than high-density foam beads is advantageous for adequate mechanical strengths of foam sheets made by compression molding.

## 4. Conclusions

The effect of the foaming pressure and temperature on cell formation in expanded TPU and the possibilities of preparing foam sheets by compression molding the prepared EPTU were studied. The effect of the structure of the foamed beads on the properties of the compression molded foam sheet was studied to obtain the following conclusion.

The TPU used in this study exhibits closed cell structures when foamed at 75–90 bar and 90–110 °C and ETPU beads having diverse foam structure with fine and/or conventional cells can be made. At higher foaming pressures, more nuclei are formed and the cell size decreases, resulting in higher expansion ratios, and the increase in the foaming temperature at a fixed pressure affects the viscoelastic property to increase the cell size and decrease the number of cells, resulting in lower expansion ratios. The possibilities of compression molding ETPU to prepare foam sheets have been confirmed, but the processing window to obtain foam sheets where the cell structure in the EPTU is not deformed during sintering of ETPU beads is very narrow. The expansion ratio of the ETPU affects the foam sheet properties with higher expansion ratios, resulting in lower hardness and thus, higher resilience. The developed cell structure also contributes to higher mechanical properties such as tensile strength and elongation at break.

## Data Availability

Data are in the authors’ possession.
